# Supplementary material for: Differentially expressed heterogeneous overdispersion genes testing for count data
Source: PLoS One. 2024 Jul 17;19(7):e0300565. doi: 10.1371/journal.pone.0300565 (PMC11253971; doi:10.1371/journal.pone.0300565)
Supplement: S1 Appendix — Numerical comparison with Limma-voom method, application on murine alveolar macrophages dataset, microglia cell experiment design, RNA-seq data preprocessing, and gene ontology analysis on Microglia cell dataset. (PDF) [file pone.0300565.s001.pdf]

Differentially Expressed Heterogeneous Overdispersion Genes Testing for Count Data

Yubai Yuan[1], Qi Xu[2], Agaz Wani [3], Jan Dahrendorff [3], Chengqi Wang[3], Arlina Shen[4], Janelle Donglasan[3], Sarah Burgan[3], Zachary Graham[3], Monica Uddin[3], Derek Wildman[3], Annie Qu[2*],

1 Department of Statistics, The Pennsylvania State University, State College, PA, USA
2 Department of Statistics, University of California Irvine, Irvine, CA,USA
3 Genomics Program, College of Public Health, University of South Florida, Tampa, FL, USA
4 University of California Berkeley, Berkeley, CA, USA

* corresponding author: aqu2@uci.edu

Appendix

Numerical comparison with Limma-voom method We perform numerical comparisons between the proposed method and the existing voom method implemented by the "Limma-voom" package" [1]. Specifically, we generate the read counts following the settings of low discrepancy in expression level in the Results section, and compare the false negative rate (FNR) and the area under the ROC curve (AUC) between the proposed method (GLM(NB)) and the "Limma" method. The results in Fig 1 and Fig 2 demonstrate that the proposed method can identify more DE genes than the "Limma" method, and achieve a similar balance between sensitivity and specificity.

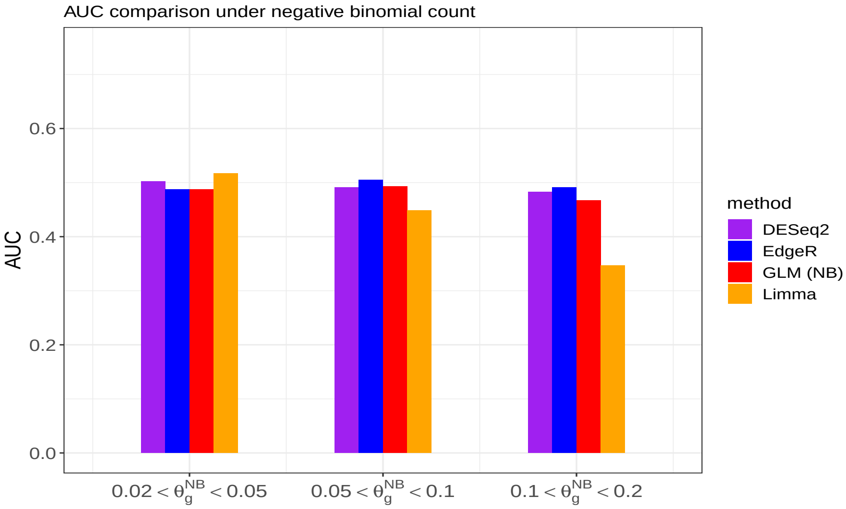

Fig 1. The false negative rate from different methods when the read counts follow the negative binomial distribution with different overdispersion levels θ_g^{NB}.

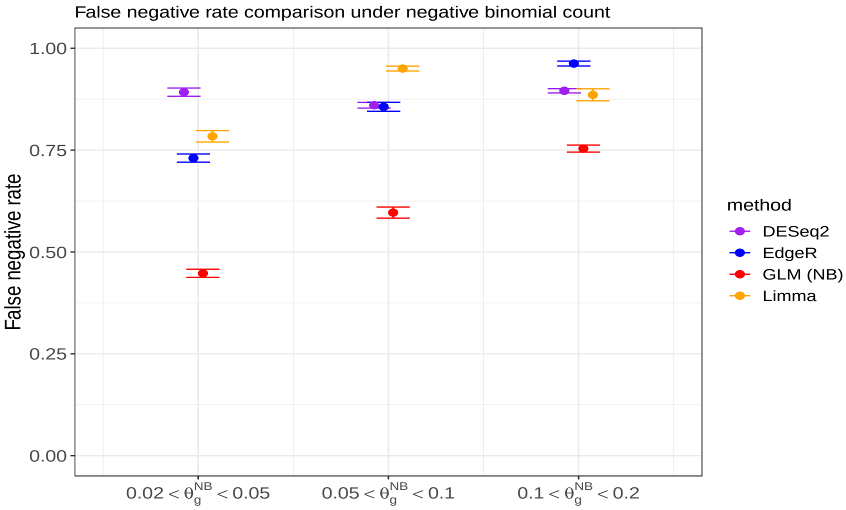

Fig 2. The area under the ROC curve from different methods when the read counts follow the negative binomial distribution with different overdispersion levels θ_g^{NB}.

Application on murine alveolar macrophages dataset We perform the proposed DEHOGT method on another benchmark RNA-seq dataset [2], and compare the differentially expressed gene detection performance with the DESeq2 and EdgeR methods. Specifically, the RNA-seq dataset is collected through the Illumina NextSeq 500 platform in a study examining transcriptional changes in alveolar macrophages after performing reperfusion in murine lung transplants. There are 43430 genes in total, each with 12 normalized read count samples: 4 samples in the control group, four samples in the group of 2 hours post-reperfusion, and four samples in the group of 24 hours post-reperfusion. We examine the DE genes between the control group and 2 hours post-reperfusion group, and between the control group and 24 hours post-reperfusion group, respectively. We then select DE genes from the above three methods, given that the false discovery rates are controlled at 0.05 and the absolute values of logfold change are larger than 1.5. The numbers of detected DE genes are listed in the following Table 1. Compared with DESeq2 and EdgeR, the proposed DEHOGT method selects more

	Treatment Pairs		
Methods	control vs 2 hour post-reperfusion	control vs 24 hour post-reperfusion	Total DE genes
DESeq2	59	153	212
EdgeR	99	192	291
DEHOGT	64	296	360

Table 1. The number of selected DE genes from murine lung transplant RNA-seq data under different treatment comparisons.

DE genes. In addition, we notice that genes *Csf3r, Gatm, Tyms Gzma, Adora2b, Fads1, Fads2, Mvk, Ube2t*, and *Haus7* are uniquely identified by the proposed method DEHOGT, and several studies [3–9] find that these genes are significantly related to the alveolar macrophages.

Microglia cell experiment design The microglial cell line HMC3 (ATCC CRL-3304, Manassas, Virginia) was used for in vitro experimentation following

successful cell line authentication and Mycoplasma testing (Genetica, Burlington, NC). HMC3 cells (passage eight) were seeded in T-25 flasks with 2 x 105 viable cells and incubated at 37°C and 5% CO2. After 24 hours, the growth medium in each T-25 was replaced with one of the following treatments: dexamethasone (1 or 0.01 µM), hydrocortisone (10 or 0.01 µM), vehicle (ethanol alcohol) or control (untreated media). Cells were incubated in treatment media for three days at 37°C and 5% CO2 and imaged daily using the Axio Vert.A1 inverted microscope (Zeiss Oberkochen, Germany). At three days post-exposure (D3), cells were collected from each flask individually, quantified on the Countess II cell counter (Invitrogen Waltham, MA), seeded at 2 x 105 viable cells/flask in new T-25 flasks with normal growth medium, and incubated for three additional days (i.e., washout period). The remaining D3 cell suspension for each flask was divided equally between two microcentrifuge tubes, pelleted and washed with PBS. One cell pellet per flask was placed in -80°C storage for future DNA extraction; the remaining cell pellet underwent RNA extraction using the RNeasy Mini Kit (QIAGEN, Hilden, Germany) protocol adapted for the QIAcube automated system (QIAGEN). On the final day of the washout period (D6), cells from each flask were imaged, collected in suspension and then quantified on the Countess II. Cell suspensions were split equally into two aliquots and then prepped for nucleic acid extraction as described for D3.

RNA samples from D3 and D6 were DNase treated (Dnase I kit; Sigma), quantified on the Qubit (RNA BR Assay Kit; Invitrogen) and scored for RNA integrity on the TapeStation (High Sensitivity RNA ScreenTape; Agilent). Library preparation was performed following the Illumina TruSeq Stranded Total RNA Library Prep Kit protocol (Illumina, San Diego, CA) with TruSeq RNA Single Indexes (Set A and B; Illumina). Library quantity and quality were assessed using the Qubit 1X dsDNA HS Assay (Invitrogen), TapeStation High Sensitivity D1000 ScreenTape (Agilent), and using the KAPA Library Quantification Kit (Roche Basel, Switzerland) for the LightCycler 96 (Roche). RNA sequencing was conducted on the NextSeq 550 (Illumina) using the High Output Kit with 76 paired-end cycles (Illumina).

RNA-seq data preprocessing The summary of processing microglia RNAseq dataset, including the workflow, FastQC setup, and genome reference, is as follows. Specifically, the raw sequencing data from the Illumina NextSeq 500 system was quality-controlled using FastQC [10], version (v) 0.12.1. The paired-end reads (150 bp read length) were trimmed removing Illumina sequencing adapters within the sequences. Additionally, low-quality reads were excluded from downstream analysis applying a Phred quality score cutoff of ¿=30. The resulting reads were aligned to the reference genome (GRCh38/HG38) using STAR v 2.7.10 [11]. Gene counts were generated using the FeatureCounts function of the R-package Subreads, v 2.0.1 [12].

Gene ontology analysis on Microglia cell dataset We perform gene ontology analysis on the genes selected from DEHOGT. In summary, our analysis shows that the selected genes are functionally relevant. From the perspective of biological processes, the selected genes are enriched in the gene ontology categories, including regulation of the immune system process, regulation of response to stress and external stimulus, and regulation of response to stimulus, which are highly relevant categories to PTSD conditions. From the perspective of cellular components, the selected genes are enriched in the categories of the ribonucleoprotein complex and the Golgi complex, which are found to be relevant in neuro-degenerative diseases formulation [13–15]. We add the detailed gene ontology analysis output in supplementary materials.

References

1. Law CW, Chen Y, Shi W, Smyth GK. voom: Precision weights unlock linear model analysis tools for RNA-seq read counts. Genome Biology. 2014;15(2):1–17.
2. Koch CM, Chiu SF, Akbarpour M, Bharat A, Ridge KM, Bartom ET, et al. A beginner's guide to analysis of RNA sequencing data. American Journal of Respiratory Cell and Molecular Biology. 2018;59(2):145–157.
3. Shi Y, Liu CH, Roberts AI, Das J, Xu G, Ren G, et al. Granulocyte-macrophage colony-stimulating factor (GM-CSF) and T-cell responses: what we do and don't know. Cell Research. 2006;16(2):126–133.
4. Yu L, Wang L, Hu G, Ren L, Qiu C, Li S, et al. Reprogramming alternative macrophage polarization by GATM-mediated endogenous creatine synthesis: A potential target for HDM-induced asthma treatment. Frontiers in Immunology. 2022;13:937331.
5. Vercammen-Grandjean A, Arnould R, Libert A, Ewalenko P, Lejeune F. Production of the effector molecule thymidine by human lung alveolar macrophages. European Journal of Cancer and Clinical Oncology. 1984;20(12):1543–1548.
6. Hwang TL, Tang MC, Kuo LM, Chang WD, Chung PJ, Chang YW, et al. YC-1 potentiates cAMP-induced CREB activation and nitric oxide production in alveolar macrophages. Toxicology and Applied Pharmacology. 2012;260(2):193–200.
7. Yan B, Fung K, Ye S, Lai PM, Wei YX, Sze KH, et al. Linoleic acid metabolism activation in macrophages promotes the clearing of intracellular Staphylococcus aureus. Chemical Science. 2022;13(42):12445–12460.
8. Lee MS, Bensinger SJ. Reprogramming cholesterol metabolism in macrophages and its role in host defense against cholesterol-dependent cytolysins. Cellular & Molecular Immunology. 2022;19(3):327–336.
9. Zhang H, Fang L, Zhu X, Wang D, Xiao S. Global analysis of ubiquitome in PRRSV-infected pulmonary alveolar macrophages. Journal of Proteomics. 2018;184:16–24.
10. Andrews S. FastQC: a quality control tool for high throughput sequence data. Available online. Retrieved May. 2010;17:2018.
11. Dobin A, Davis CA, Schlesinger F, Drenkow J, Zaleski C, Jha S, et al. STAR: ultrafast universal RNA-seq aligner. Bioinformatics. 2013;29(1):15–21.
12. Liao Y, Smyth GK, Shi W. The R package Rsubread is easier, faster, cheaper and better for alignment and quantification of RNA sequencing reads. Nucleic Acids Research. 2019;47(8):e47–e47.
13. Liu J, Huang Y, Li T, Jiang Z, Zeng L, Hu Z. The role of the Golgi apparatus in disease. International Journal of Molecular Medicine. 2021;47(4):1–1.
14. Martínez-Menárguez JÁ, Tomás M, Martínez-Martínez N, Martínez-Alonso E. Golgi fragmentation in neurodegenerative diseases: is there a common cause? Cells. 2019;8(7):748.
15. Ule J. Ribonucleoprotein complexes in neurologic diseases. Current Opinion in Neurobiology. 2008;18(5):516–523.
